# A highly efficient sorbitol dehydrogenase from *Gluconobacter oxydans* G624 and improvement of its stability through immobilization

**DOI:** 10.1038/srep33438

**Published:** 2016-09-16

**Authors:** Tae-Su Kim, Sanjay K. S. Patel, Chandrabose Selvaraj, Woo-Suk Jung, Cheol-Ho Pan, Yun Chan Kang, Jung-Kul Lee

**Affiliations:** 1 Department of Chemical Engineering, Konkuk University, Seoul 05029, Korea; 2 Systems Biotechnology Research Center, KIST Gangneung Institute of Natural Products, 25451, Republic of Korea; 3 Department of Materials Science and Engineering, Korea University, Seoul 02841, Republic of Korea

## Abstract

A sorbitol dehydrogenase (GoSLDH) from *Gluconobacter oxydans* G624 (*G. oxydans* G624) was expressed in *Escherichia coli* BL21(DE3)-CodonPlus RIL. The complete 1455-bp codon-optimized gene was amplified, expressed, and thoroughly characterized for the first time. GoSLDH exhibited *K*_m_ and *k*_cat_ values of 38.9 mM and 3820 s^−1^ toward L-sorbitol, respectively. The enzyme exhibited high preference for NADP^+^ (vs. only 2.5% relative activity with NAD^+^). GoSLDH sequencing, structure analyses, and biochemical studies, suggested that it belongs to the NADP^+^-dependent polyol-specific long-chain sorbitol dehydrogenase family. GoSLDH is the first fully characterized SLDH to date, and it is distinguished from other L-sorbose-producing enzymes by its high activity and substrate specificity. Isothermal titration calorimetry showed that the protein binds more strongly to D-sorbitol than other L-sorbose-producing enzymes, and substrate docking analysis confirmed a higher turnover rate. The high oxidation potential of GoSLDH for D-sorbitol was confirmed by cyclovoltametric analysis. Further, stability of GoSLDH significantly improved (up to 13.6-fold) after cross-linking of immobilized enzyme on silica nanoparticles and retained 62.8% residual activity after 10 cycles of reuse. Therefore, immobilized GoSLDH may be useful for L-sorbose production from D-sorbitol.

L-Sorbose is a unique sugar that is used extensively as a primary precursor for the biosynthesis of L-ascorbic acid in a two-step fermentation of D-sorbitol to L-sorbose and L-sorbose to 2-keto-l-gulonic acid^1^,^2^. It is used in the synthesis of 1-deoxygalactonojirimycin, a glycosidase inhibitor^3^, and as a starting material for producing L-tagatose^4^. *Gluconobacter* and *Acetobacter* are well known for the effective conversion of D-sorbitol into L-sorbose through regio-controlled D-sorbitol dehydrogenation^5^,^6^. *G. oxydans* is preferred in the production of L-sorbose from D-sorbitol via biotransformation. In addition, it has the ability to partially oxidize glucose and glycerol to gluconic acid and dihydroxypropanone, respectively^7^. These reactions are primarily associated with membrane-bound D-sorbitol dehydrogenase (SLDH). Hence, the bioconversion of D-sorbitol to L-sorbose is largely limited by SLDH due to its instability and difficulty in inducing expression^7^. Although the expression of the SLDH gene has been evaluated in *Pseudomonas putida* IF03738^8^, SLDH from *G. oxydans* (GoSLDH) might be further characterized, engineered, or heterologously expressed in a suitable host to solve these problems. In addition, the requirement of a co-factor for the enzymatic bioconversion process is an important concern, and a minimum amount of cofactor should be used in real industrial processes. Therefore, a co-factor recycling system can be implemented for efficient and economic biotransformation^9^,^10^.

In addition to soluble expression, the stability of GoSLDH is a major concern. The recombinant GoSLDH obtained through its heterologous expression in *P. putida* was very unstable and lost its activity completely within 3 days of storage at −20 °C^8^. Therefore, stabilization strategies can be adapted to improve the enzyme properties to enhance its performance in industrial applications^11^,^12^,^13^. Immobilization of the enzyme is an approach to provide stability and allow recovery from the reaction mixture. Various immobilization methods have been employed for enzymes based on physical, covalent, cross-linking or affinity interactions. Among these methods, covalent immobilization resulted in higher stability through strong attachments^14^,^15^,^16^,^17^,^18^,^19^,^20^,^21^,^22^,^23^,^24^. Nanoparticle-based support for the immobilization of enzymes is widely used due to the advantages of nanoparticles such as availability in different sizes and compositions, high surface area, and a robust nature. In contrast, the biocompatibility of nanoparticles is a primary concern due to their toxic nature^15^,^18^,^20^,^23^,^24^. Silica-based nanoparticles are considered highly suitable for the immobilization of various types of enzymes as a result of their unique properties, including biocompatibility, resistance towards solvents, and microbial attacks.

Previously, 15 kb and 40 kb of assemblies containing the SLDH gene were cloned in *Escherichia coli* DH-1 and XL1-Blue MRA, respectively^8^. However, expression and characterization of the recombinant SLDH protein has not been reported. In this study, we heterologously expressed and fully characterized a recombinant polyol-specific long-chain GoSLDH. Based on biochemical and homology modeling data, GoSLDH was found to exhibit higher catalytic efficiency than any other L-sorbose-producing enzymes^25^,^26^,^27^,^28^,^29^,^30^. Further, stability of the SLDH was improved through immobilization on silica (SiO_2_) nanoparticles, resulting in high reusability. There is a need to produce a catalytically efficient and stable SLDH to improve the production of L-sorbose from D-sorbitol because of its broad industrial applications. GoSLDH is a promising candidate for the efficient production of L-sorbose from D-sorbitol.

## Results and Discussion

L-Sorbose is recognized as a suitable intermediate in the industrial manufacturing of value-added chemicals such as vitamin C, 1-deoxygalactonojirimycin, and L-tagatose^1^,^2^,^3^,^4^. Bacterial fermentation has become the sole method for L-sorbose production^31^. Therefore, a catalytically efficient and stable SLDH is required to improve the bioconversion of D-sorbitol to L-sorbose^25^,^26^,^27^,^28^,^29^,^30^. Based on the conserved catalytic motif (KXXXXHXXH) in polyol-specific long-chain dehydrogenase, GoSLDH can be grouped into the subfamily of polyol-specific long-chain dehydrogenases^32^. A previous study reported the fermentative production of L-sorbose from D-sorbitol by *G. oxydans* G624^31^. Here, *G. oxydans* G624 showed SLDH activity (43.2 U/mL) and a 20% conversion yield from D-sorbitol to L-sorbose at 18 h of fermentation^31^. However, the conversion rate was low, and this process remained suboptimal for high yields. In addition, the concentration of D-sorbitol was inhibitory at >10% (w/v). Thus, the results showed its limited potential for industrial application. To overcome these problems, we successfully cloned, expressed, and characterized the *sldh* gene from *G. oxydans* G624. Although the properties of L-sorbose-producing microbial enzymes have been determined, as listed in Table 1, GoSLDH, the first SLDH to be thoroughly characterized, exhibited higher activity than the previously characterized L-sorbose-producing enzymes, including SLDH and mannitol dehydrogenase (MDH)^25^,^26^,^27^,^28^,^29^,^30^.

### *GoSLDH* characterization

The cosmid sequence of *G. oxydans* G624 *sldh* was utilized to study the BAA99414.1 protein as described previously^8^. The identified GoSLDH had 32–82% sequence identity with polyol-specific long-chain dehydrogenase family enzymes. A comparative alignment of 17 sequences is presented in Fig. S1. Further, the catalytic region of *Pseudomonas fluorescens* MDH (PfMDH) was identified as the previously suggested conserved sequence (KXXXXNXXH) of polyol-specific long-chain dehydrogenase (Fig. S1). This GoSLDH alignment (Q9KWR5) revealed the presence of three highly conserved residues, Lys294, Asn299, and His302, that have been found in other known polyol-specific long-chain dehydrogenases^32^. In addition, GoSLDH possessed the active site residues of Asp190, Val228, Lys294, and Asp299. Based on these studies, we considered GoSLDH a polyol-specific long-chain dehydrogenase in *G. oxydans* G624. Based on the conserved catalytic motif (KXXXXHXXH) for polyol-specific long-chain dehydrogenase, GoSLDH can be grouped as a subfamily of polyol-specific long-chain dehydrogenases^30^,^32^.

### Expression and characterization of the GoSLDH enzyme

#### Codon optimization and expression of the GoSLDH gene

**Figure 1:**
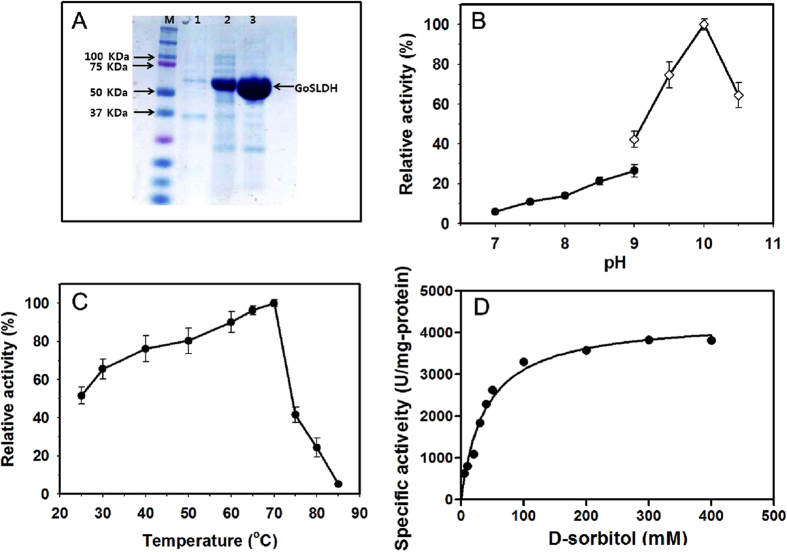
Purification and characterization of GoSLDH. (**A**) Determination of the molecular mass of *Gluconobacter oxydans* G624 GoSLDH by SDS-PAGE. SDS-PAGE analysis of GoSLDH expressed in *E. coli* BL21 (DE3)-CodonPlus RIL was carried out on a 12% gel. Lane M: molecular standard marker, insoluble protein (Lane 1), soluble protein (Lane 2) and purified protein (Lane 3) GoSLDH. (**B**) Effect of pH on the activity of purified GoSLDH. Filled circles in Tris-HCl (100 mM) buffer (pH 7.0–9.0) and filled diamonds in glycine-NaOH (100 mM) buffer (pH 9.0–10.5). (**C**) Effect of temperature on the activity of purified GoSLDH. (**D**) Effect of substrate concentration on the activity of purified GoSLDH.

A codon-optimized *Gosldh* sequence of 1,455 bp containing 6× His tag was synthesized. The synthesized *Gosldh* gene was cloned into pET28a, and the recombinant plasmids containing the *Gosldh* gene were transformed into the *E. coli* BL21 (DE3)-CodonPlus RIL host strain. SDS-PAGE analysis was performed to evaluate recombinant protein expression as shown in Fig. 1A. The obtained GoSLDH had a molecular mass of 53.64 kDa. Further, an activity assay confirmed that GoSLDH was specifically produced as a target protein. The optimum pH and temperature for the purified GoSLDH were determined (Fig. 1B,C). Maximum activity was observed at pH 10 (100 mM glycine-NaOH buffer) and 70 °C. GoSLDH was subjected to dialysis with 10 mM EDTA after purification in order to analyze the effects of metal ions. This EDTA treatment did not affect the initial enzyme activity. K^+^, Ba^2+^, Ca^2+^, Cu^2+^, and Zn^2+^ metal ions resulted in up to 30% inhibition of GoSLDH (Table S1). Mg^2+^, Co^2+^, and Mn^2+^ ions showed no stimulatory influence on GoSLDH activity under similar conditions.

#### Substrate and coenzyme specificity

Substrate specificity was evaluated using D-sorbitol, mannitol, xylitol, ribitol (adonitol), myo-inositol, glycerol, and D/L-arabinitol (200 mM) as substrates. The observed results suggested that GoSLDH is highly specific towards D-sorbitol, mannitol, and D-arabinitol (Table S2). No activity was detected with L-arabinitol, xylitol, ribitol, myo-inositol, and glycerol. On the other hand, GoSLDH exhibited a preference for NADP^+^ over NAD^+^ as a coenzyme. Measurement of activity with D-sorbitol as a substrate suggested that GoSLDH is exclusively an NADP^+^-dependent enzyme, resulting in only 2.5% relative activity with NAD^+^. The recombinant GoSLDH exhibited high activity with D-sorbitol as a substrate, in addition to mannitol and arabinitol among various substrates. These results suggest that recombinant GoSLDH is highly specific and selective towards these substrates at an alkaline optimum pH.

#### Kinetic parameters

Purified GoSLDH showed the highest specific activity, 3570 U/mg, with D-sorbitol using NADP^+^ as a coenzyme. Initial velocities were determined in glycine-NaOH buffer (pH 10) with D-sorbitol concentrations increasing from 5 to 400 mM under standard assay conditions (Fig. 1D). The *K*_m_ values of 88.2 μM and 38.9 mM for NADP^+^ and D-sorbitol, respectively, and turnover rate (*k*_*cat*_) of 3820 s^−1^ were obtained via a nonlinear regression analysis. The *k*_*cat*_/*K*_*m*_ value for GoSLDH against D-sorbitol was 98.1 mM^−1^s^−1^. GoSLDH showed the highest catalytic efficiency with D-sorbitol among a number of L-sorbose-producing enzymes (Table 1).

#### Substrate docking

The reference PDB ID of the GoSLDH crystal structure is 5ITG. The structure of GoSLDH and PfMDH bound with D-sorbitol lacked sufficient detail to reveal precisely how it bound. To understand the binding conformation of D-sorbitol along with GoSLDH and PfMDH, molecular docking was performed. Molecular docking assesses the interactions of the protein with the ligand using scoring and energy parameters. The binding orientation of D-sorbitol with GoSLDH and PfMDH was similar, but the scoring and interactions showed a major difference. D-Sorbitol showed a −8.68 kcal/mol docking score and −56.30 kcal/mol docking energy with GoSLDH (Table S3; Fig. S2). In the case of PfMDH with D-sorbitol, the docking score was −6.519 kcal/mol and the docking energy was −41.17 Kcal/mol. A hydrogen-bonding network was formed between the hydrogen bond donors and acceptors in both GoSLDH and PfMDH. D-Sorbitol formed strong interactions of five hydrogen bonds with GoSLDH, with Asn190, Lys294, and Leu191 playing the roles of interacting amino acids (Fig. S3A). In comparison with GoSLDH, PfMDH shows weak bonding of only three hydrogen bonds with Asn191 and Lys381. In both, the Asn and Lys residues showed similar interactions, but Leu191 gave additional support to GoSLDH (Fig. S3A,B). In addition to the hydrogen bonding network, the amino acids Met227, Val287, Ile295, and Phe384 showed hydrophobic bonding with the D-sorbitol in GoSLDH. In PfMDH, Leu192, Phe385, and Tyr136 showed hydrophobic bonding between the protein and ligand (Fig. S2).

**Figure 2:**
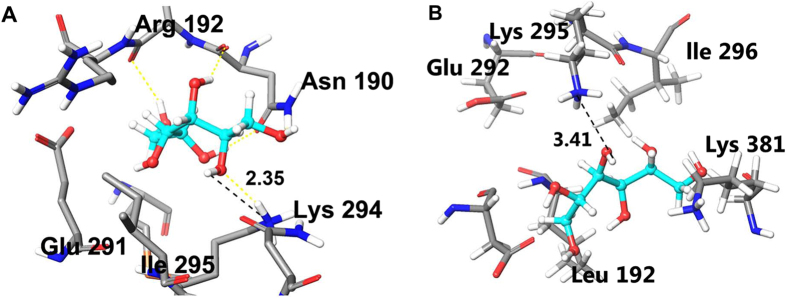
Substrate docking of GoSLDH and PfMDH with NAD(P)^+^ and D-sorbitol. (**A**) Distance between the Lys294 (N atom) and D-sorbitol (H atom) in GoSLDH. (**B**) Distance between the Lys295 (N atom) and D-sorbitol (H atom) in PfMDH. All other atoms are colored according to standard coloring. Amino acid residues are shown by the tube model, whereas bound D-sorbitol is represented by the ball-and-stick model. Active-site coordinating bond lengths are shown in Å. Images were generated using Maestro 10.2.

The reaction mechanism for the conversion of mannitol to fructose by PfMDH was reported previously^33^. In this process, the hydroxyl group at C2 is oxidized via sequential abstractions of protons from the sugar O_2_ and transfer of this proton to bulk solvent, followed by the hydride transfer from the sugar (C2) to nicotinamide (C4). Finally, the Lys residue at position 295 acts as a proton acceptor. Therefore, the distance between PfMDH Lys295 and O_2_ hydrogen of the substrate (mannitol or D-sorbitol) is mechanistically important for the abstraction of protons for oxidation^33^. Our docking results were cross-checked with the reaction mechanism of GoSLDH and PfMDH. The distances between the protein and ligand atoms are tabulated in Table S3. The mean distance value of D-sorbitol with GoSLDH was 1.9 Å, whereas the average distance of D-sorbitol with PfMDH was 2.08 Å. In GoSLDH, the distance between the N atom (K294) and H atom was analyzed. The distance between Lys294 and the O_2_ hydrogen atom of D-sorbitol was 2.35 Å (Fig. 2), which favors close contact. This was also seen in PfMDH, which showed a distance of 3.41 Å between the Lys295 N-atom and H-atoms (Fig. 2). This result suggests that the GoSLDH distance is shorter than that of PfMDH, which may allow easier abstraction of protons from D-sorbitol O_2_ hydrogens. Overall, the interaction pattern, distance matrix, and scoring parameters suggest that GoSLDH shows stronger binding with D-sorbitol than does PfMDH. This may be the reason for the higher turnover rate of D-sorbitol by purified GoSLDH (3820 S^−1^) than by PfMDH (12.8 S^−1^).

### Binding energy calculation

Molecular docking with a related post-scoring approach (MM-GBSA) was performed for D-sorbitol-bound GoSLDH and PfMDH. The MM-GBSA results for the free energy of binding prediction are presented in Table S3. The calculated free energy of D-sorbitol bound to the GoSLDH complex was −37.46 kcal/mol, and the value with PfMDH was −32.61 kcal/mol. The MM-GBSA scoring results generally significantly correlate with the activity determined experimentally, although the process is more computationally demanding. Based on the components of the binding-free energy, the major favorable contributors to ligand binding are nonpolar solvation (ΔGsolv SA) and van der Waals terms. However, polar solvation (ΔGsolv GB) opposes binding as suggested previously^34^. Thus, GoSLDH appears to have a strong binding affinity with D-sorbitol and can be used for L-sorbose production. In comparison, energy intake was more efficient for GoSLDH than for PfMDH, indicating that the docking interactions favor the distance between the C2-OH (D-sorbitol) and nitrogen (Lys294), which utilizes the high energy yield better than PfMDH. These results are consistent with the turnover rates of GoSLDH (*k*_*cat*_ = 3820 S^−1^) and PfMDH (*k*_*cat*_ = 12.8 S^−1^). Overall, the interaction pattern, distance matrix, and scoring parameters suggest that GoSLDH binds more strongly to D-sorbitol than does PfMDH.

### CV measurements

**Figure 3:**
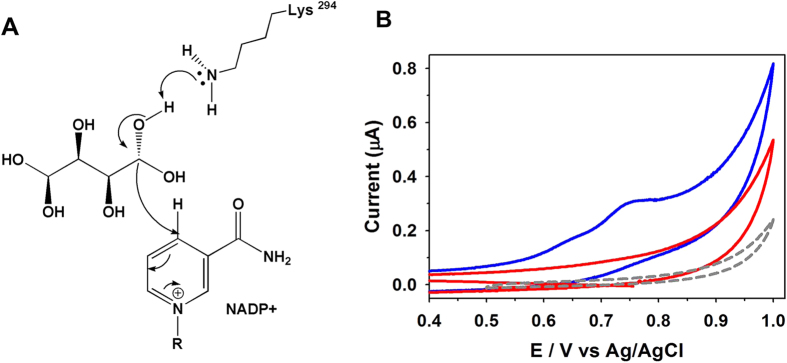
Electrochemical property of GoSLDH. (**A**) Reaction mechanism of GoSLDH. (**B**) Electrocatalytic voltammograms for D-sorbitol oxidation by dehydrogenases. The oxidation of D-sorbitol by GoSLDH (blue line), PfMDH (red line), and blank (gray dot line) are shown.

Given the shorter distance between Lys294 and the O_2_ hydrogen of the substrate, it seems that GoSLDH could abstract a proton more efficiently than PfMDH (Fig. 2). After proton abstraction, the hydride is transferred from D-sorbitol to NADP^+^ with the electron flowing to the positively charged nitrogen of NADP^+^ to serve as an electron sink (Fig. 3A). As a result, D-sorbitol is oxidized. The D-sorbitol oxidation activities of GoSLDH and PfMDH were compared based on electron transfer capability using CV. The oxidation current peak using D-sorbitol was found to be 0.35 μA at a potential of 0.76 V (Fig. 3B). Under the same conditions, PfMDH exhibited negligible oxidation of D-sorbitol with a peak current of 0.01 μA. Based on these results, GoSLDH is adequate to efficiently electrocatalytically oxidize D-sorbitol, leading to a higher operating potential than PfMDH. This result suggests that GoSLDH can more efficiently oxidize D-sorbitol than PfMDH.

### ITC analysis

**Figure 4:**
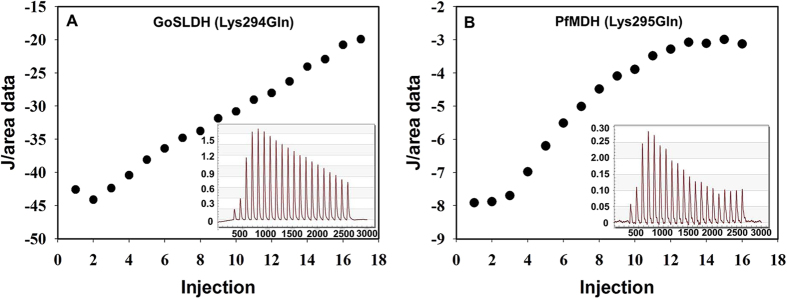
Thermodynamic contributions to D-sorbitol binding by GoSLDH (Lys294Gln) and PfMDH (Lys195Gln) determined by isothermal titration calorimetry. (**A**) Purified GoSLDH. (**B**) Purified PfMDH. The inset graph shows thermograms of GoSLDH (Lys294Gln) and PfMDH (Lys195Gln).

Binding affinities between D-sorbitol and GoSLDH or PfMDH were determined by ITC. The *K*_*d*_ (dissociation constant) values were determined for inactive GoSLDH (Lys294Q) and PfMDH (Lys295Q), which were lacking the catalytic residues for substrate conversion during the titration experiments. The binding curves indicated that the heat emitted per mole of titrant as a function of the molar ratio of total ligand to total enzyme was similar to a previous analysis (Fig. 4)^35^. The GoSLDH affinity (Lys294Gln, *K*_d_ = 96 μM) towards D-sorbitol was 2.9-fold higher than that of PfMDH (Lys295Gln, *K*_d_ = 278 μM). These bindings were driven by the energy contribution ΔG (Gibbs free energy) values of −22.88 and −20.29 kJ mol for GoSLDH (Lys294Gln) and PfMDH (Lys295Gln), respectively. In addition, PfMDH (Lys295Gln) binding to D-sorbitol showed an increase in ΔG of 2.59 kJ mol. The lower ΔG value of GoSLDH (Lys294Gln) was likely due to a high binding affinity between GoSLDH and D-sorbitol. This clearly shows that GoSLDH exhibits better binding with D-sorbitol than does PfMDH. These results are consistent with the kinetic parameters (*K*_*m*_) of GoSLDH and PfMDH.

### Immobilization of GoSLDH

**Figure 5:**
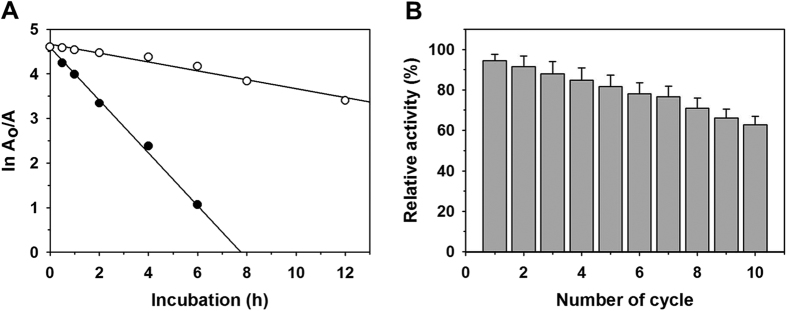
Stability of free (filled circle) and immobilized GoSLDH (open circle) at 25 °C (**A**) and reusability of immobilized GoSLDH on SiO2 particles (**B**).

The purified recombinant GoSLDH was very unstable, with a t_1/2_ of 70 min at 25 °C, although it was more stable than the GoSLDHs reported previously^8^,^27^. Thus, the covalent immobilization of GoSLDH onto SiO_2_ nanoparticles was performed to improve its stability. Here, the amino groups of amino acids such as lysine present on the surface of GoSLDH react with the glutaraldehyde activated SiO_2_ nanoparticles to form covalent bonds during immobilization at pH 7^21^. The IY and IE of GoSLDH immobilized on different silica nanoparticles were in the ranges of 40.4–71.2% and 53.5–76.7%, respectively (Table 2). These variations in IY and IE were primarily associated with the properties of the silica particles^16^,^21^. Immobilization of GoSLDH onto 4830HT SiO_2_ particles resulted in the highest IY and IE values of 71.2% and 76.7%, respectively. This efficient enzyme loading and IE on SiO_2_ (80 nm) was mainly due to the large surface area of particles (440 m^2^/g). Further, modified SiO_2_ particles with carbodiimide and cyanogen groups were evaluated for the immobilization of GoSLDH^20^. These modified particles resulted in lower IY and IE values, in the ranges of 53.2–57.7 and 54.7–67.4%, than the particles activated by glutaraldehyde (Fig. S4A). These results suggested that both IY and IE are significantly dependent on the methods of immobilization. The maximum loading of GoSLDH was 207 mg/g of support on glutaraldehyde functionalized SiO_2_ particles (Fig. S4B). The presence of additional observed peaks at 1,600–1,800 cm^−1^ in the FTIR spectra of GoSLDH on particles, corresponding to the amide bonds and -N=C=O and C=C stretches as described previously^21^, confirmed that the enzyme was successfully immobilized (Fig. S5). Additional peaks at 3,444, 1,630, and 1,083 cm^−1^ were correlated with -OH stretching, C-O bending, and Si-O-Si stretching, respectively^21^. The residual activity of purified GoSLDH followed similar trends over a pH range of 7–10, and the stability slightly increased with an increase in enzyme concentration from 0.1 to 1.0 U/mL (Fig. S6). The thermal dissociation constants (*k*_d_) for free and immobilized GoSLDH were 0.59 and 0.10 per h at 25 °C, respectively (Fig. 5A). These values indicate that both free and immobilized GoSLDH activities were decreased with an increase in incubation time. Here, the significantly lower *k*_d_ value represents the high stability of the immobilized GoSLDH (Table S4). The *t*_1/2_ value of 70 min for free GoSLDH was significantly increased to 420 min after immobilization at 25 °C, and a 6-fold increase in stability was observed after immobilization. To improve the stability of the immobilized enzyme, we then performed the cross-linking of immobilized GoSLDH by glutaraldehyde (0.5%) as described previously^21^,^23^. After cross-linking, the stability improved 13.6-fold compared to the free enzyme (Table S4). Here, the significant stabilization of the enzyme after immobilization might be primarily associated with favorable interactions between the enzyme and support. Reusability is a key parameter of an immobilized enzyme for its efficient application. After 10 cycles of reuse, the immobilized GoSLDH retained about 62.8% of residual activity (Fig. 5B). Here, the high reusability of immobilized GoSLDH might be primarily associated with its higher stability^21^. Similarly, after 10 days of storage at 4 °C, immobilized GoSLDH retained approximately 74.6% of residual activity, whereas the free enzyme retained less than 5% of activity under the same conditions (Fig. S7). In contrast, a previous report suggested that recombinant GoSLDH expressed in *P. putida* was very unstable, even at −20 °C^8^.

### L-Sorbose production by GoSLDH

D-Sorbitol, a naturally abundant and commercially available polyol (1.8 €/kg; chemical market report 2009) was used as the starting material to produce L-sorbose. Purified GoSLDH was employed to oxidize D-sorbitol into L-sorbose. Further, the rate of either D-sorbitol utilization or L-sorbose formation was analyzed. The maximum bioconversion to L-sorbose reached 47% after 3 h of reaction (Fig. S8). The product was confirmed as L-sorbose by the HPLC retention time of pure L-sorbose of 9.2 min. Absence of additional peaks suggested that there was not formation of by-products during the course of the reaction (Fig. S8). The D-sorbitol oxidation product was recovered and polarimetrically analyzed. It was confirmed with an [α]^D^ of −43.4°, matching that of a L-sorbose standard (Table S5).

## Conclusions

This study reports the detailed characterization of recombinant GoSLDH, showing the highest activity towards D-sorbitol ever reported. Further, the high activity of GoSLDH was analyzed in a structural analysis together with CV and ITC studies. These observations suggested that recombinant GoSLDH is more catalytically efficient than other SLDH or MDH due to the close proximity of D-sorbitol to the catalytic residue, along with high binding affinity. The stability of GoSLDH was significantly improved by its immobilization on the SiO_2_ nanoparticles. This recombinant GoSLDH with high catalytic activity can be used for efficient L-sorbose production.

## Materials and Methods

### Plasmids and reagents

The Ex-Taq DNA polymerase, a genomic DNA extraction kit, and PCR reagents were purchased from Promega (Madison, USA). Restriction enzymes were purchased from New England Biolabs (MA, USA)^36^. The expression vector, plasmid isolation kit, and NiNTA superflow column were obtained from Qiagen (Hilden, Germany)^36^. Oligonucleotide primers were obtained from Bioneer (Daejeon, South Korea). Chemicals used in assays were purchased from Sigma-Aldrich (St. Louis, MO, USA). Nanoparticles were obtained from Nanostructured and Amorphous Materials (Houston, TX, USA) as described previously^21^.

### Bacterial strains and culture conditions

The genomic sequence and SLDH protein sequence of *G. oxydans* G624 were accessed in the NCBI database (www.ncbi.nlm.nih.gov). *E. coli* DH-5*α* and *E. coli* BL21 (DE3)-CodonPlus RIL strains were used as hosts for transformation of plasmids and expression, respectively, as described previously^36^. These strains were grown in LB medium supplemented with 50 μg mL^−1^ of kanamycin at 37 °C and were maintained at 4 °C.

### *sldh* gene synthesis and expression vector construction

The codon-optimized *sldh* gene (1.458 kb) was synthesized according to the DNA sequence of the polyol-specific long-chain dehydrogenase from *G. oxydans* G624 (GenBank accession number: KU535615). The optimized *sldh* gene sequence fused with a 6× His tag was named *Gosldh*. The *Gosldh* gene fragment, flanked with *Nde*I and *Xho*I restriction enzymes sites at the 5′ and 3′ ends, was synthesized by GeneScript (Piscataway, NJ, USA). The codon-optimized *Gosldh* was digested by *Nde*I-*Xho*I and ligated into the pET-28(a) vector for the expression of *Gosldh*, and the respective N-terminal His6-tag protein was expressed.

### Expression and purification of protein

The *E. coli* BL21 (DE3)-CodonPlus RIL strain containing *Gosldh* fused with a 6× His tag was grown in LB medium (50 mL) supplemented with 50 μg mL^−1^ kanamycin at 16 °C under shaking (250 rpm). Further, isopropyl-β-d-galactopyranoside was added to a 0.4 mM final concentration (OD_600_ of the culture, approximately 0.3–0.4), and incubation with shaking was continued for 20 h. Cells were centrifuged at 4,000 rpm for 12 min and 4 °C as described previously^21^. The resulting pellets were resuspended in pH 10.0 glycine-NaOH buffer (100 mM). Further, cells were sonicated for 10 min at 4 °C, and lysates were centrifuged at 14,000× *g* for 20 min at 4 °C to remove cell debris as described previously^21^. The cell-free extract of crude GoSLDH was purified on a Ni-NTA Superflow Column as described previously^36^,^37^,^38^. Enzyme samples were analyzed by sodium dodecyl sulfate-polyacrylamide gel electrophoresis (SDS-PAGE) and stained with SimplyBlue™ SafeStain^39^,^40^. Protein concentrations were determined by the Bradford method as described previously^41^,^42^,^43^.

### Determination of molecular mass

GoSLDH molecular mass was calculated using a protein molecular weight standard and a Bio-Sil SEC-250 column (300 × 7.8 mm, Bio-Rad) on an HPLC system (Shimadzu, Kyoto, Japan). Here, the mobile phase (pH 6.8) consisted of Na_2_HPO_4_ (50 mM), NaH_2_PO_4_ (50 mM), NaCl (50 mM), and NaN_3_ (10 mM), and the flow rate was maintained at 1.0 mL/min. Further, the quaternary structures of the protein were analyzed through the comparison of molecular weights observed by HPLC and SDS–PAGE (Fig. 1A).

### Enzyme assays

Activity was determined spectrophotometrically through the measurements of rate formation of NADH (ε = 5.62 mM^−1^ cm^−1^) or NADPH (ε = 5.12 mM^−1^ cm^−1^) at 340 nm at room temperature. A 1 mL standard reaction mixture was used in the assay containing cofactor NAD(P)^+^ (0.5 mM) and substrate (20–200 mM) along with enzyme in pH 10 glycine-NaOH buffer (100 mM). One unit of GoSLDH activity was defined as the enzyme amount needed to catalyze the formation of 1 μmol NAD(P)H per minute.

### Determining the optimum temperature and pH values

To determine the optimum pH, the enzyme activity was assessed in the range of 7.0–10.5 in the following buffers (100 mM): Tris-HCl (pH 7.0–9.0) and glycine-NaOH (pH 9.0–10.5). Similarly, the optimal temperature vale was determined by analyzing the enzyme activity at 20–85 °C.

### Effect of metal ions

Initially, purified GoSLDH was dialyzed against glycine-NaOH buffer (100 mM, pH 10) with EDTA (10 mM) for 24 h 4 °C and thereafter with pure glycine-NaOH. GoSLDH was concentrated by ultrafiltration using an Amicon-Ultra15 filter (Amicon Corp., Danvers, MA). Under standard assay conditions, the resulting enzyme was used to study of the effect of different metal ions (MnCl_2,_ KCl, BaCl_2,_ CaCl_2_, CuSO_4_, ZnCl_2_, CoCl_2_, and MgCl_2_) at 0.5 mM concentrations on GoSLDH activity. The observed activities were compared with those of the free enzyme under the same conditions (absence of metal ions).

### Kinetic parameters

The GoSLDH kinetic parameters were examined at 25 °C using substrate (D-sorbitol) and co-factor (NADP^+^) concentrations in the ranges of 5–400 mM and 0.1–2.0 mM in glycine-NaOH (100 mM) buffer at the optimum pH value. Kinetic parameters (apparent *K*_*m*_ and *V*_*max*_) were determined using nonlinear regression-fitting analyses of the data in GraphPad Prism 5 software (Grappa Software, Inc., CA, USA) as described previously^20^. The kinetic data are presented as the averages of statistically relevant triplicate measurements with standard deviations^16^.

### Grid generation and molecular docking

The crystal structures of GoSLDH (PDB ID: 5ITG) and PfMDH (PDB ID: 1LJ8) were used for docking analysis. Both optimized structures were minimized until the average Root-Mean-Square-Deviation (RMSD) of the non-hydrogen atoms reached 0.3 Å as described previously^44^. D-sorbitol (3-D structure) was retrieved from the PubChem database, and the ligand structure was prepared using LigPrep v3.5. The bond orders, length, and angle were fixed and minimized with the Optimized Potentials for Liquid Simulations (OPLS-2005) force field as described previously^45^. After the protein and ligand structure was minimized and setup for the molecular modeling calculations through GLIDE grid generation, the grid was generated for both proteins^46^. Sitemap v3.6 was used to identify the five highest ranked potential binding sites for the prepared structures of GoSLDH and PfMDH using high accuracy and a restrictive hydrophobicity definition^47^. The XYZ axes of the grid box were set around the predicted sites, and to soften the receptor potential for non-polar interactions, we scaled the van der Waals radii of receptor atoms by 2.00 Å with a partial charge cut-off of 0.25. Successful grids were docked with D-sorbitol using the Extra Precision (XP) mode in Glide, and the results were analyzed as described previously^34^,^48^. Glide Emodel combines the Glidescore, coulombic, van der Waals, and strain energy of the ligand as described previously^49^.

### Binding energy calculation

The free energy of binding was calculated for both docked complexes with D-sorbitol using the Prime/MM-GBSA method as described previously^34^. It was performed using the OPLS-2005 force field and GBSA continuum model in Prime version 3.0 (Schrodinger) as described previously^50^,^51^.

The binding free energy, ΔG_bind,_ was obtained using the following equations as described previously^52^:


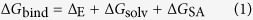



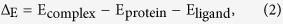


where E_ligand_, E_protein_, and E_complex_ are the minimized energies of the inhibitor, protein, and protein-inhibitor complex, respectively.


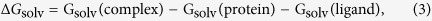


where G_solv_ (ligand), G_solv (_protein), and G_solv_ (complex) are the solvation free energies of the inhibitor, protein, and complex, respectively.


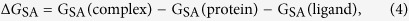


where G_SA_ (ligand), G_SA_ (protein), and G_SA_ (complex) are the surface area energies for the ligand, protein, and complex, respectively^53^.

### Site-directed mutagenesis

Mutagenesis was performed through Stratagene QuikChange site-directed mutagenesis kit (La Jolla, CA, USA). Recombinant plasmids pET 28(a)-*GoSLDH* and pET-28a-*PfMDH* containing codon-optimized *GoSLDH* and *PfMDH* were used as DNA templates. The plasmids were transformed in *E. coli* BL21-CodonPlus (DE3)-RIL. Kanamycin- and chloramphenicol-resistant colonies were selected and used for protein expression. Mutants of GoSLDH and PfMDH were purified and subsequently concentrated via centrifugation (3,000 × *g*) using ultrafiltration spin columns (VivaSpin 20) obtained from Sartorius Stedim Biotech (Göttingen, Germany) at 4 °C.

### Electrochemical measurements

CV analysis was performed to compare the oxidation potential of GoSLDH and PfMDH using an SP-150 potentiostat (BioLogic, USA). A three-electrode setup of Ag/AgCl, glassy carbon electrode (GCE), and platinum wire as a reference, counter, and working electrode were used for analysis, respectively, as described previously^23^. Enzyme samples (5 μl) at a ratio of 9:1 with nafion (5%) were fixed on the GCE and dried at 4 °C for 12 h. The prepared electrodes were used for the CV determination of D-sorbitol oxidation in buffer (pH 10) containing NADP^+^ and NAD^+^ as cofactors (2 mM) for GoSLDH and PfMDH, respectively.

### Isothermal titration calorimetry

ITC measurements were carried out using a Nano ITC (TA Instruments, DE, USA). In a typical ITC experiment, D-sorbitol (1 mM) was titrated with the GoSLDH Lys294Gln (3.7 mg/mL) and PfMDH Lys295Gln (3.7 mg/mL) mutants as described previously^35^. Titrations were carried out at 25 °C with 20 injections (2.4 μL) of inactive mutant enzymes or their variant solutions. All solutions were filtered, degassed to avoid bubble formation, and equilibrated to the appropriate temperature before starting the experiment. The observed data were analyzed using Nano Analyze (TA 100 Instruments) and Origin software as described previously^35^. Enthalpy of binding was determined on the basis of three titrations each for GoSLDH (Lys294Gln) and PfMDH mutants (Lys295Gln). The intrinsic molar enthalpy change (ΔHa), binding stoichiometry (n), binding constant (K_a_), Gibbs free energy (ΔG^b^), and ΔS were obtained as described previously^35^.

### Immobilization of the purified GoSLDH

SiO_2_ particles were functionalized by glutaraldehyde, 1-cyclohexyl-3-(2-morpholinoethyl) carbodiimide metho-*p*-toluenesulfonate, and cyanogen bromide to produce aldehyde, carbodiimide, and cyanogen groups on the particle surfaces for the covalent immobilization of GoSLDH as described previously^16^,^20^. The various modified SiO_2_ particles (10 mg) were mixed with 1 mL of purified GoSLDH (1.0 mg protein), and incubation was performed for 24 h (4 °C) under shaking (150 rpm) conditions. Further, immobilized enzymes on the particles were recovered through centrifugation and subsequently washed three times with buffer (100 mM potassium phosphate, pH 7.0). The Bradford method was used for protein concentration measurements in the washed solution as described previously^16^. Immobilized enzyme residual activity was determined under standard assay conditions. The IE and IY were calculated as described previously^20^. The loading of enzyme on the particles was examined at different enzyme concentrations (50–800 mg/g of support). The stability at 25 °C and storage stability at 4 °C of immobilized GoSLDH were studied in glycine-NaOH buffer at the optimum pH. The reusability of GoSLDH immobilized on SiO_2_ nanoparticles was examined at 25 °C over 10 cycles under assay conditions. For each cycle, the reusability was assessed by monitoring the rate of NADPH formation under standard assay conditions until stable absorbance at 340 nm was reached. The initial activity of the immobilized enzyme was considered as 100%. After each cycle, the immobilized enzyme was removed by centrifugation as described previously^21^ and then washed with buffer and used for the second cycle. Each assay reaction was carried out three times.

### Sorbose production and analysis

Reaction mixture (1 mL) containing sorbitol (50 mM) and NADP (50 mM) in pH 10 glycine-NaOH buffer (100 mM) was used for to obtain the product. The prepared reaction mixture was incubated for 2 h at 30 °C. Samples were withdrawn for the product analysis at various intervals using the cysteine carbazole sulfuric method at an absorbance of 560 nm^54^. Further, products were confirmed by HPLC (Ultimate 3000, USA) as described earlier^21^. Retention times of 11 min and 9.8 min were observed for D-sorbitol and L-sorbose, respectively. In addition, the D- or L-configuration of the obtained products (D- or L-sugars) was monitored by their rotation angles in a quartz cell at 25 °C with deuterium lamp emissions at 589 nm using pure standards (D- and L-sorbose)^55^.

## Additional Information

**How to cite this article**: Kim, T.-S. *et al.* A highly efficient sorbitol dehydrogenase from *Gluconobacter oxydans* G624 and improvement of its stability through immobilization. *Sci. Rep.* **6**, 33438; doi: 10.1038/srep33438 (2016).

## Supplementary Material

Supplementary Information

## Figures and Tables

**Table 1:** Biochemical and kinetic properties of D-sorbitol oxidizing polyol dehydrogenases from various organisms.

Organism	Mr (kDa)	Quaternary structure	Optimum pH (oxidation)	Optimum Temp. (°C)	km (mM)	*k*_*cat*_ (s^−1^)	*k*_*cat*_*/K*_*m*_ (mM^−1^ s^−1^)	Reference
*Acetobacter suboxydans*	28	Dimer	8.5	30	127	19.7	0.15	29
*Aspergillus fumigatus*	58	Monomer	10.0	25	680	60	0.09	30
*Pseudomonas fluorescens* (PfMDH)	54	Monomer	10.0	25	426	12.8	0.03	25
*Gluconobacter oxydans*	29	Dimer	8.5	25	125	2.57	0.02	26
*Gluconobacter frateurii* THD32	135	Multi-subunits	4.5	25	20.4	211	10.35	27
*Gluconobacter oxydans* M5	80	Monomer	6.0	30	16.8	199	11.85	28
*G. oxydans* G624 (GoSLDH)	54	Dimer	10	70	38.9	3820	98.1	This study

**Table 2:** Effect of different metal ions on the activity of recombinant GoSLDH.

Silica particles	Surface area (m^2^/g)	Immobilization yield (%)	Immobilization efficiency (%)
4806SF	5	40.4	53.5
4860MR	160	53.1	62.4
4830HT	440	71.2	76.0

## References

[b1] Gao L., Zhou J., Liu J., Du G. & Chen J. Draft genome sequence of *Gluconobacter oxydans* WSH-003, a strain that is extremely tolerant of saccharides and alditols. J. Bacteriol. 194, 4455–4456 (2012). 22843589 10.1128/JB.00837-12 PMC3416238

[b2] Zhu Y., Liu J., Du G., Zhou J. & Chen J. Sporulation and spore stability of *Bacillus megaterium* enhance *Ketogulonigenium vulgare* propagation and 2-keto-L-gulonic acid biosynthesis. Bioresour. Technol. 107, 399–404 (2012). 22257860 10.1016/j.biortech.2011.12.080

[b3] Huwig A., Emmel S., Jäkel G. & Giffhorn F. Enzymatic synthesis of L-tagatose from galactitol with galactitol dehydrogenase from *Rhodobacter sphaeroides* D. Carbohyd. Res. 305, 337–339 (1997).

[b4] Leang K. *et al.* A novel enzymatic approach to the massproduction of L-galactose from L-sorbose. J. Biosci. Bioeng. 97, 383–388 (2004). 16233647 10.1016/S1389-1723(04)70223-6

[b5] Zebiri I., Balieu S., Guilleret A., Reynaud R. & Haudrechy A. The Chemistry of L-Sorbose. Eur. J. Org. Chem. 2011, 2905–2910 (2011).

[b6] Macauley-Patrick S., McNeil B. & Harvey L. A. By-product formation in the D-sorbitol to L-sorbose biotransformation by *Gluconobacter suboxydans* ATCC 621 in batch and continuous cultures. Process Biochem. 40, 2113–2122 (2005).

[b7] Xu S., Wang X., Du G., Zhou J. & Chen J. Enhanced production of L-sorbose from D-sorbitol by improving the mRNA abundance of sorbitol dehydrogenase in *Gluconobacter oxydans* WSH003. Microb. Cell Fact. 13, 146 (2014). 25323199 10.1186/s12934-014-0146-8 PMC4205284

[b8] Shibata T. *et al.* Cloning of a gene for D-sorbitol dehydrogenase from *Gluconobacter oxydans* G624 and expression of the gene in *Pseudomonas putida* IFO3738. J. Biosci. Bioeng. 89, 463–468 (2000). 16232778 10.1016/s1389-1723(00)89097-0

[b9] Liu W. & Wang P. Cofactor regeneration for sustainable enzymatic biosynthesis. Biotechnol. Adv. 25, 369–384 (2007). 17459647 10.1016/j.biotechadv.2007.03.002

[b10] Berenguer-Murcia A. & Fernandez-Lafuente R. New trends in the recycling of NAD(P)H for the design of sustainable asymmetric reductions catalyzed by dehydrogenases. Curr. Org. Chem. 14, 1000–1021 (2010).

[b11] Bolivar J. M. *et al.* Stabilization of a formate dehydrogenase by covalent immobilization on highly activated glyoxyl-agarose supports. Biomacromolecules 7, 669–673 (2006). 16529396 10.1021/bm050947z

[b12] Bolivar J. M. *et al.* Immobilization-stabilization of a new recombinant glutamate dehydrogenase from *Thermus thermophilus*. Appl. Microbiol. Biotechnol. 80, 49–58 (2008). 18545996 10.1007/s00253-008-1521-3

[b13] Fernandez-Lafuente R. Stabilization of multimeric enzymes: Strategies to prevent subunit dissociation. Enzyme Microb. Technol. 45, 405–418 (2009).

[b14] Mateo C., Palomo J. M., Fernandez-Lorente G., Guisan J. M. & Fernandez-Lafuente R. Improvement of enzyme activity, stability and selectivity via immobilization techniques. Enzyme Microb. Technol. 40, 1451–1463 (2007).

[b15] Garcia-Galan C., Berenguer-Murcia A., Fernandez-Lafuente R. & Rodrigues R. C. Potential of different enzyme immobilization strategies to improve enzyme performance. Adv. Synth. Catal. 353, 2885–2904 (2011).

[b16] Zhang Y. W., Tiwari M. K., Jeya M. & Lee J. K. Covalent immobilization of recombinant *Rhizobium etli* CFN42 xylitol dehydrogenase onto modified silica nanoparticles. Appl. Microbiol. Biotechnol. 90, 499–507 (2011). 21246353 10.1007/s00253-011-3094-9

[b17] Liese A. & Hilterhaus L. Evaluation of immobilized enzymes for industrial applications. Chem. Soc. Rev. 42, 6236–6249 (2013). 23446771 10.1039/c3cs35511j

[b18] Rodrigues R. C., Ortiz C., Berenguer-Murcia A., Torres R. & Fernández-Lafuente R. Modifying enzyme activity and selectivity by immobilization. Chem. Soc. Rev. 42, 6290–6307 (2013). 23059445 10.1039/c2cs35231a

[b19] Barbosa O. *et al.* Glutaraldehyde in bio-catalysts design: A useful crosslinker and a versatile tool in enzyme immobilization. RSC Adv. 4, 1583–1600 (2014).

[b20] Patel S. K. *et al.* Immobilization of laccase on SiO_2_ nanocarriers improves its stability and reusability. J. Microbiol. Biotechnol. 24, 639–647 (2014). 24509251 10.4014/jmb.1401.01025

[b21] Singh R. K., Tiwari M. K., Singh R., Haw J. R. & Lee J. K. Immobilization of L-arabinitol dehydrogenase on aldehyde-functionalized silicon oxide nanoparticles for L-xylulose production. Appl. Microbiol. Biotechnol. 98, 1095–1104 (2014). 24193245 10.1007/s00253-013-5209-y

[b22] Barbosa O. *et al.* Strategies for the one-step immobilization-purification of enzymes as industrial biocatalysts. Biotechnol. Adv. 33, 435–456 (2015). 25777494 10.1016/j.biotechadv.2015.03.006

[b23] Patel S. K. S., Choi S. H., Kang Y. C. & Lee J.-K. Large-scale aerosol-assisted synthesis of biofriendly Fe_2_O_3_ yolk–shell particles: a promising support for enzyme immobilization. Nanoscale 8, 6728–6738 (2016). 26952722 10.1039/c6nr00346j

[b24] Verma M. L., Puri M. & Barrow C. J. Recent trends in nanomaterials immobilised enzymes for biofuel production. Crit. Rev. Biotechnol. 36, 108–119 (2016). 25017196 10.3109/07388551.2014.928811

[b25] Slatner M., Nidetzky B. & Kulbe K. D. Kinetic study of the catalytic mechanism of mannitol dehydrogenase from *Pseudomonas fluorescens*. Biochemistry 38, 10489–10498 (1999). 10441145 10.1021/bi990327g

[b26] Cheng H., Jiang N., Shen A. & Feng Y. Molecular cloning and functional expression of D-arabitol dehydrogenase gene from *Gluconobacter oxydans* in Escherichia coli. FEMS Microbiol. Lett. 252, 35–42 (2005). 16165327 10.1016/j.femsle.2005.08.023

[b27] Toyama H., Soemphol W., Moonmangmee D., Adachi O. & Matsushita K. Molecular properties of membrane-bound FAD-containing D-sorbitol dehydrogenase from thermotolerant *Gluconobacter frateurii* isolated from Thailand. Biosci. Biotechnol. Biochem. 69, 1120–1129 (2005). 15973043 10.1271/bbb.69.1120

[b28] Yang X. P., Wei L. J., Ye J. B., Yin B. & Wei D. Z. A pyrroloquinoline quinine-dependent membrane-bound D-sorbitol dehydrogenase from *Gluconobacter oxydans* exhibits an ordered Bi Bi reaction mechanism. Arch. Biochem. Biophys. 477, 206–210 (2008). 18407824 10.1016/j.abb.2008.03.030

[b29] Cheng H., Li Z., Jiang N. & Deng Z. Cloning, purification and characterization of an NAD-Dependent D-Arabitol dehydrogenase from acetic acid bacterium. Acetobacter suboxydans. Protein J. 28, 263–272 (2009). 19629658 10.1007/s10930-009-9191-2

[b30] Krahulec S., Armao G. C., Klimacek M. & Nidetzky B. Enzymes of mannitol metabolism in the human pathogenic fungus *Aspergillus fumigatus* – kinetic properties of mannitol-1-phosphate 5-dehydrogenase and mannitol 2-dehydrogenase, and their physiological implications. FEBS J. 278, 1264–1276 (2011). 21299839 10.1111/j.1742-4658.2011.08047.x

[b31] Saito Y. *et al.* Cloning of genes coding for L-sorbose and L-sorbosone dehydrogenases from *Gluconobacter oxydans* and microbial production of 2-keto-L-gulonate, a precursor of L-ascorbic acid, in a recombinant *G. oxydans* strain. Appl. Environ. Microbiol. 63, 454–460 (1997). 9023923 10.1128/aem.63.2.454-460.1997 PMC168335

[b32] Klimacek M. & Nidetzky B. A catalytic consensus motif for D-mannitol 2-dehydrogenase, a member of a polyol-specific long-chain dehydrogenase family, revealed by kinetic characterization of site-directed mutants of the enzyme from *Pseudomonas fluorescens*. Biochem. J. 367, 13–18 (2002). 12175334 10.1042/BJ20020932 PMC1222881

[b33] Kavanagh K. L., Klimacek M., Nidetzky B. & Wilson D. K. Crystal structure of *Pseudomonas fluorescens* mannitol 2-dehydrogenase binary and ternary complexes. Specificity and catalytic mechanism. J. Biol. Chem. 277, 43433–43442 (2002). 12196534 10.1074/jbc.M206914200

[b34] Selvaraj C., Priya R. B., Lee J. K. & Singh S. K. Mechanistic insights of SrtA-LPXTG blockers targeting the transpeptidase mechanism in *Streptococcus mutans*. RSC Adv. 5, 100498–100510 (2015).

[b35] Prabhu P. *et al.* Structure-based studies on the metal binding of two-metal-dependent sugar isomerases. FEBS J. 281, 3446–3459 (2014). 24925069 10.1111/febs.12872

[b36] Zhang Y. W. *et al.* Cloning and characterization of a thermostable H_2_O-forming NADH oxidase from *Lactobacillus rhamnosus*. Enzyme Microb. Technol. 50, 255–262 (2012). 22418266 10.1016/j.enzmictec.2012.01.009

[b37] Ohk S. O. *et al.* Heterologous expression and characterization of CYP61A1 from dandruff-causing Malassezia globosa. Protein Expres. Purif. 114, 89–94 (2015). 10.1016/j.pep.2015.07.002 26160660

[b38] Rajesh T. *et al.* Phosphorylation of chloramphenicol by a recombinant protein Yhr2 from *Streptomyces avermitilis* MA4680. Bioorg. Med. Chem. Lett. 23, 3614–3619 (2013). 23659856 10.1016/j.bmcl.2013.04.015

[b39] Lee K. M. *et al.* Enhanced enzymatic hydrolysis of rice straw by removal of phenolic compounds using a novel laccase from yeast *Yarrowia lipolytica*. Bioresour. Technol. 123, 636–645 (2012). 22960123 10.1016/j.biortech.2012.07.066

[b40] Singh R. K. *et al.* Molecular cloning and characterization of a GH11 endoxylanase from *Chaetomium globosum*, and its use in enzymatic pretreatment of biomass. Appl. Microbiol. Biotechnol. 97, 7205–7214 (2013). 23184220 10.1007/s00253-012-4577-z

[b41] Bradford M. M. A rapid and sensitive method for the quantitation of microgram quantities of protein utilizing the principle of protein-dye binding. Anal. Biochem. 72, 248–254 (1976). 942051 10.1016/0003-2697(76)90527-3

[b42] Choi S. H. *et al.* A brief method for preparation of gintonin-enriched fraction from ginseng. J. Ginseng. Res. 39, 398–405 (2015). 26869834 10.1016/j.jgr.2015.05.002 PMC4593782

[b43] Kim H. R., Kim K. W., Kim B. M., Cho M. L. & Lee S. H. The effect of vascular endothelial growth factor on osteoclastogenesis in rheumatoid arthritis. PloS One 10, e0124909 (2015). 25894998 10.1371/journal.pone.0124909 PMC4404365

[b44] Selvaraj C., Singh S. K., Tripathi S. K., Reddy K. K. & Rama M. In silico screening of indinavir-based compounds targeting proteolytic activity in HIV PR: binding pocket fit approach. Med. Chem. Res. 21, 4060–4068 (2012).

[b45] Muralidharan A. R. *et al.* Structure-Based Virtual Screening and Biological Evaluation of a Calpain Inhibitor for Prevention of Selenite-Induced Cataractogenesis in an *in Vitro* System. J. Chem.Inf. Model. 55, 1686–1697 (2015). 26270943 10.1021/acs.jcim.5b00092

[b46] Tripathi S. K., Soundarya R. N., Singh P. & Singh S. K. Comparative Analysis of Various Electrostatic Potentials on Docking Precision Against Cyclin-Dependent Kinase 2 Protein: A Multiple Docking Approach. Chem. Biol. Drug. Des. 85, 107–118 (2015). 24923208 10.1111/cbdd.12376

[b47] Selvaraj C., Omer A., Singh P. & Singh S. K. Molecular insights of protein contour recognition with ligand pharmacophoric sites through combinatorial library design and MD simulation in validating HTLV-1 PR inhibitors. Mol. Biosyst. 11, 178–189 (2015). 25335799 10.1039/c4mb00486h

[b48] Friesner R. A. *et al.* Extra precision glide: Docking and scoring incorporating a model of hydrophobic enclosure for protein-ligand complexes. J. Med. Chem. 49, 6177–6196 (2006). 17034125 10.1021/jm051256o

[b49] Halgren T. A. *et al.* Glide: a new approach for rapid, accurate docking and scoring. 2. Enrichment factors in database screening. J. Med. Chem. 47, 1750–1759 (2004). 15027866 10.1021/jm030644s

[b50] Du J. *et al.* Molecular Modeling Study of Checkpoint Kinase 1 Inhibitors by Multiple Docking Strategies and Prime/MM-GBSA Calculation. J. Comput. Chem. 32, 2800–2809 (2011). 21717478 10.1002/jcc.21859

[b51] Lyne P. D., Lamb M. L. & Saeh J. C. Accurate prediction of the relative potencies of members of a series of kinase inhibitors using molecular docking and MM-GBSA scoring. J. Med. Chem. 49, 4805–4808 (2006). 16884290 10.1021/jm060522a

[b52] Selvaraj C., Sivakamavalli J., Baskaralingam V. & Singh S. K. Virtual screening of LPXTG competitive SrtA inhibitors targeting signal transduction mechanism in *Bacillus antracis*: a combined experimental and theoretical study. J. Recept. Signal Transduct. Res. 34, 221–232 (2014). 24490975 10.3109/10799893.2013.876044

[b53] Greenidge P. A., Kramer C., Mozziconacci J. C. & Wolf R. M. MM/GBSA Binding Energy Prediction on the PDBbind Data Set: Successes, Failures, and Directions for Further Improvement. J. Chem. Inf. Model. 53, 201–209 (2013). 23268595 10.1021/ci300425v

[b54] Dische Z. & Borenfreund E. A new spectrophotometric method for the detection and determination of keto sugars and trioses. J. Biol. Chem. 192, 583–587 (1951). 14907652

[b55] Jagtap S. S., Singh R., Kang Y. C., Zhao H. & Lee J. K. Cloning and characterization of a galactitol 2-dehydrogenase from *Rhizobium legumenosarum* and its application in D-tagatose production. Enzyme Microb. Technol. 58–59, 44–51 (2014). 10.1016/j.enzmictec.2014.02.012 24731824

