# Supplementary material for: A highly efficient sorbitol dehydrogenase from Gluconobacter oxydans G624 and improvement of its stability through immobilization
Source: Sci Rep. 2016 Sep 16;6:33438. doi: 10.1038/srep33438 (PMC5025769; doi:10.1038/srep33438)
Supplement: Supplementary Information [file srep33438-s1.doc]

***Supplementary Information***

**A highly efficient sorbitol dehydrogenase from *Gluconobacter oxydans* G624 and improvement of its stability through immobilization**

**Tae-Su Kim^1+^, Sanjay K. S. Patel^1+^, Chandrabose Selvaraj^1^, Woo-Suk Jung^2^, Cheol-Ho Pan^2^, Yun Chan Kang^3^*, Jung-Kul Lee^1^***

^1^Department of Chemical Engineering, Konkuk University, Seoul 05029, Korea

^2^Functional Food Center, KIST Gangneung, 25451, Republic of Korea

^3^Department of Materials Science and Engineering, Korea University, Seoul 02841, Republic of Korea

^+^These authors equally contributed to this study

^*^Author for correspondence: jkrhee@konkuk.ac.kr

^*^Author for correspondence: yckang@korea.ac.kr

**
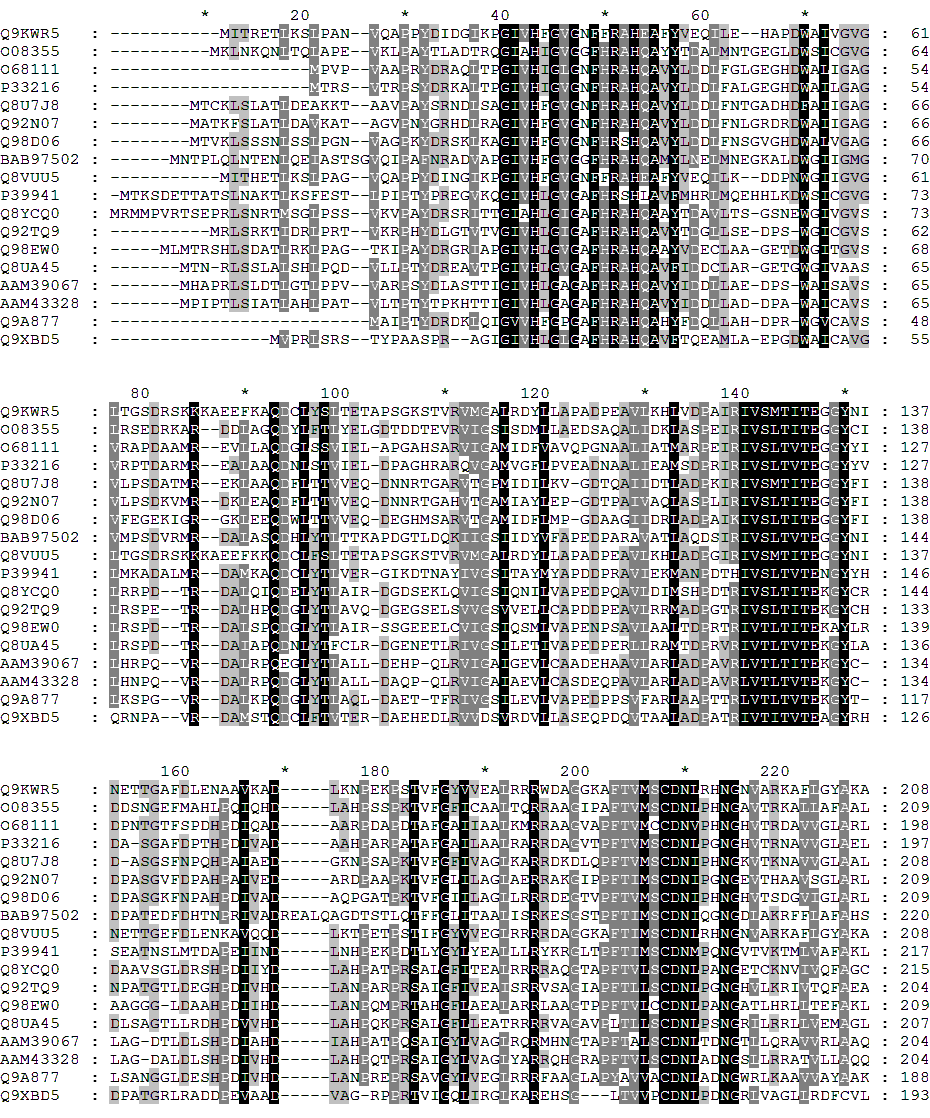
**


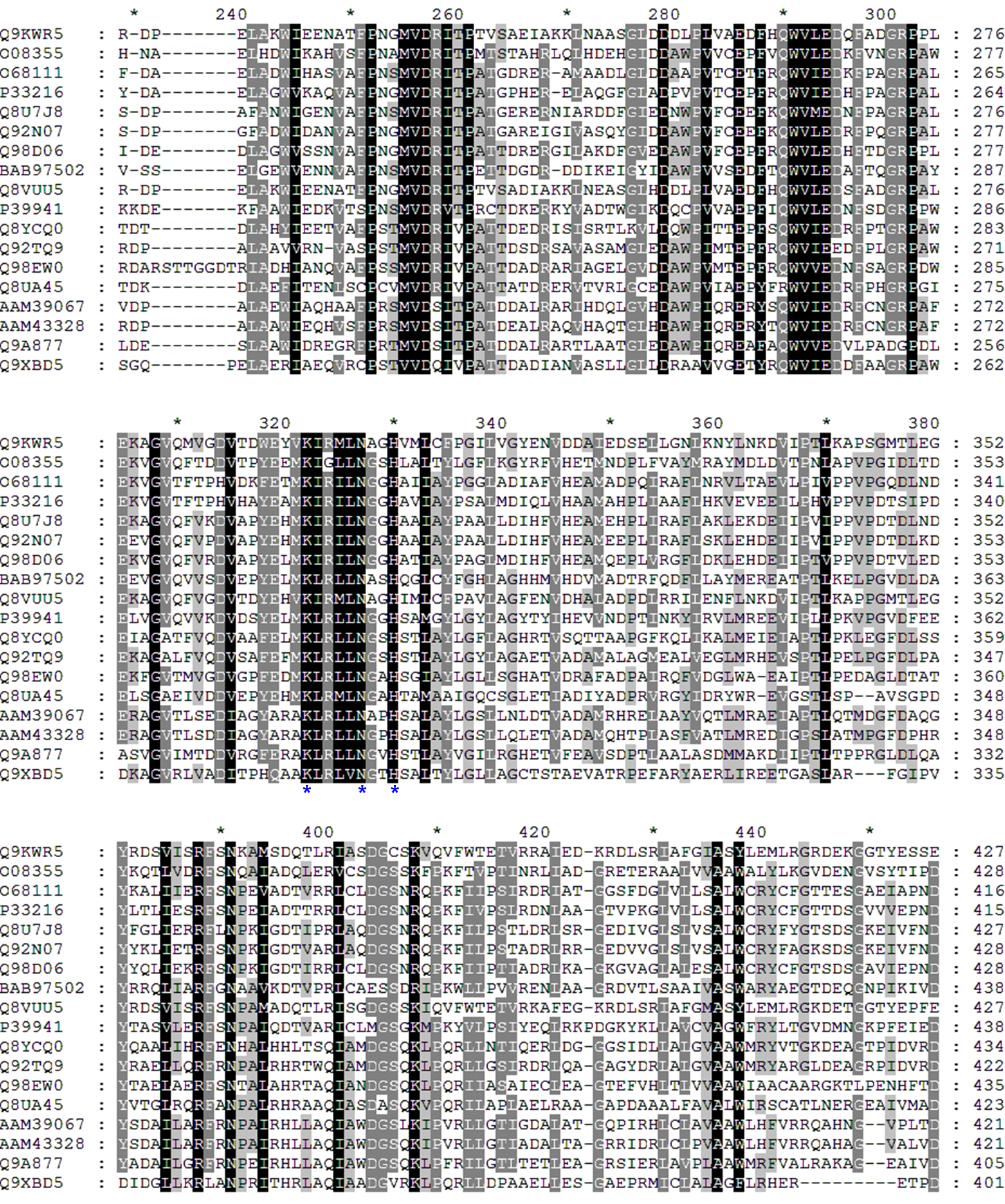


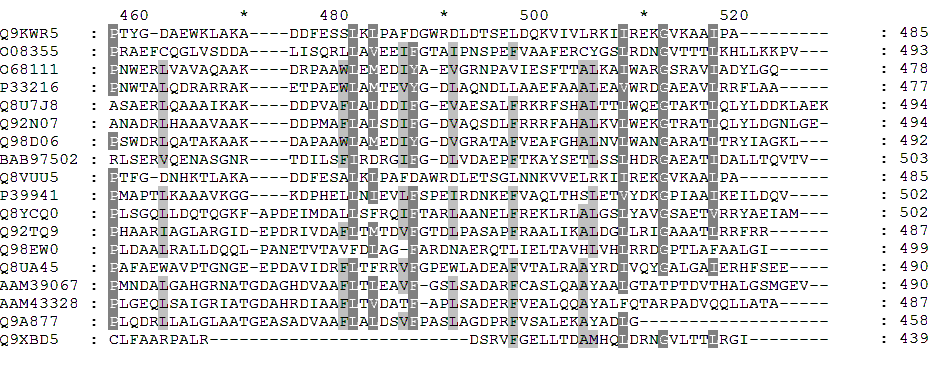


**Figure S1.** Identification of a conserved KXXXNXXH motif by an alignment of multiple polyol-specific long-chain dehydrogenase sequences. Nineteen representative PSLDR members are shown in the aligned protein sequences. Q9KWR5, d-sorbitol dehydrogenase from *Gluconobacter oxydans*; O08355, d-mannitol 2-dehydrogenase from *Pseudomonas* *fluorescens*; O68111, d-mannitol 2-dehydrogenase from *Rhodobacter capsulatus*; P33216, mannitol 2-dehydrogenase from *Rhodobacter sphaeroides*; Q8U7J8, mannitol 2-dehydrogenase from *Agrobacterium tumefaciens* str. C58; Q92N07, mannitol 2-dehydrogenase from *Sinorhizobium meliloti*; Q98D06, mannitol dehydrogenase from *Mesorhizobium loti*; BAB97502, mannitol 2-dehydrogenase from *Corynebacterium glutamicum ATCC 13032*; Q8VUU5, l-sorbose reductase from *Gluconobacter oxydans*; P39941, mannitol dehydrogenase from *Saccharomyces cerevisiae S288c*; Q8YCQ0, mannonate oxidoreductase from *Brucella melitensis*; Q92TQ9, d-mannonate oxidoreductase from *Rhizobium meliloti*; Q98EW0, mannitol dehydrogenase from *Mesorhizobium loti*; Q8UA45, mannitol dehydrogenase from *Agrobacterium tumefaciens* str. C58; AAM39067, mannitol dehydrogenase from *Xanthomonas axonopodis* pv. *citri* str. 306; AAM43328, mannitol dehydrogenase from *Xanthomonas campestris* pv. *campestris* str. ATCC 33913; Q9A877, mannitol 2-dehydrogenase from *Caulobacter crescentus*; Q9XBD5, mannitol dehydrogenase from *Amycolatopsis orientalis*. Conserved residues are marked with * (blue)

**A**


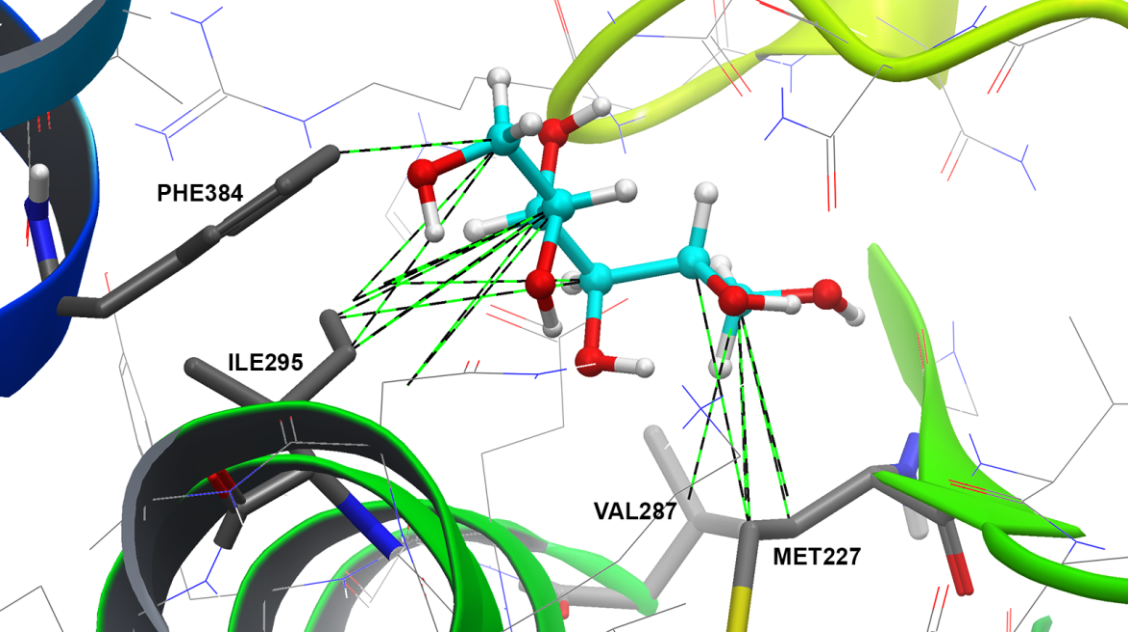


**B**


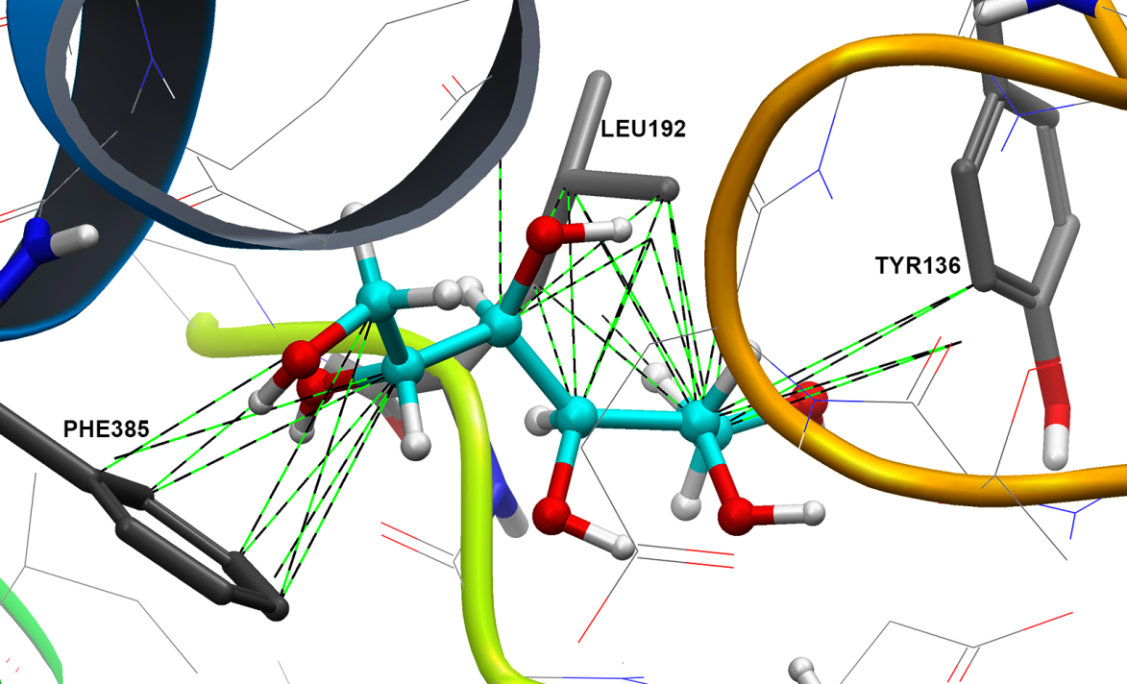


**Figure S2.** Hydrophobic interactions between the d-sorbitol and (A) GoSLDH and (B) PfMDH.

**A**

**
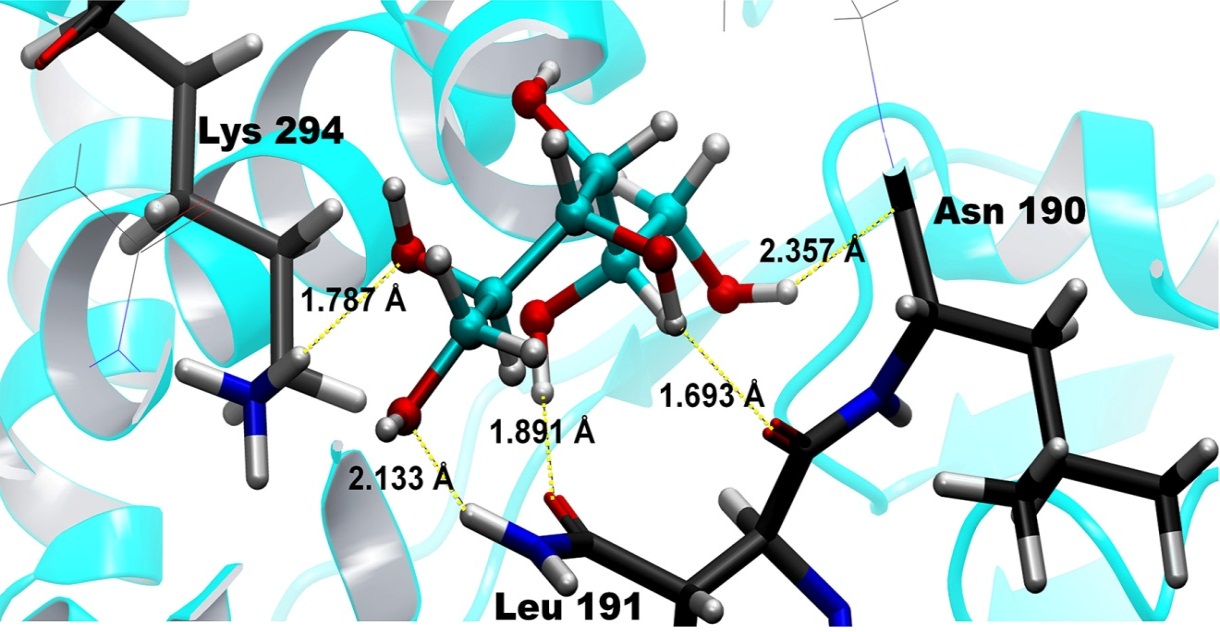
**

**B**

**
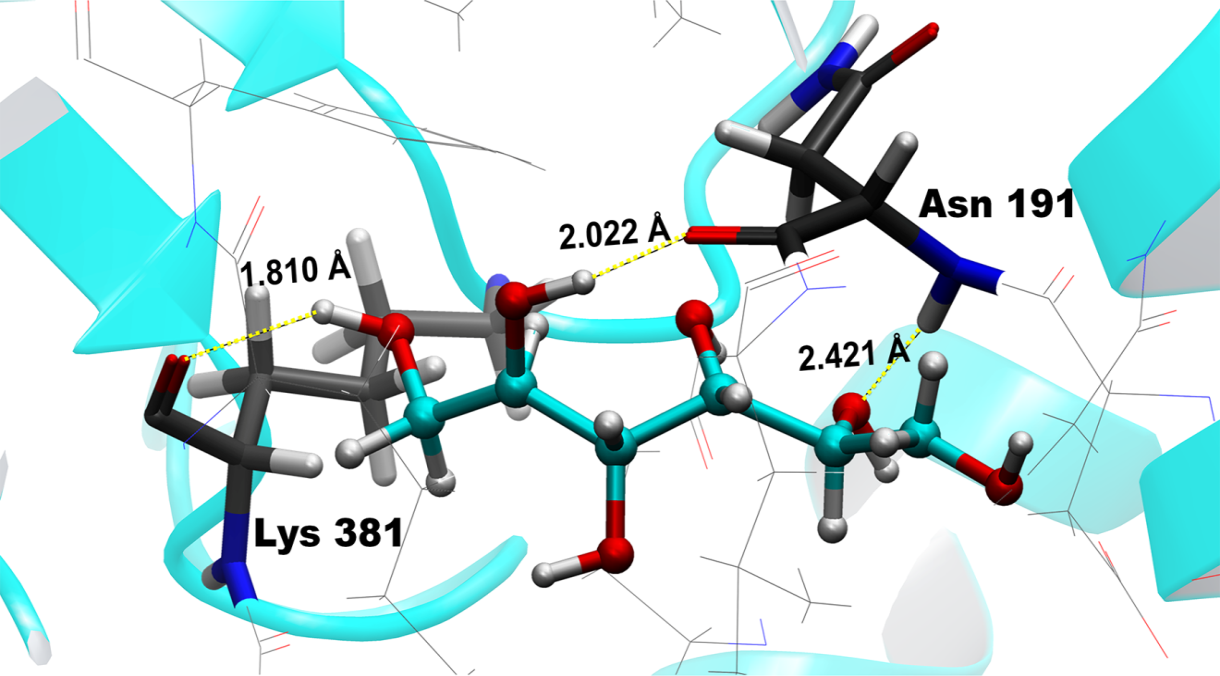
**

**Figure S3.** Molecular docking of d-sorbitol to the GoSLDH (A) and PfMDH (B) active-site pocket. The catalytic residues of GoSLDH and PfMDH are shown in gray color carbon. The hydrogen bonds are shown in yellow discontinuous lines.

**
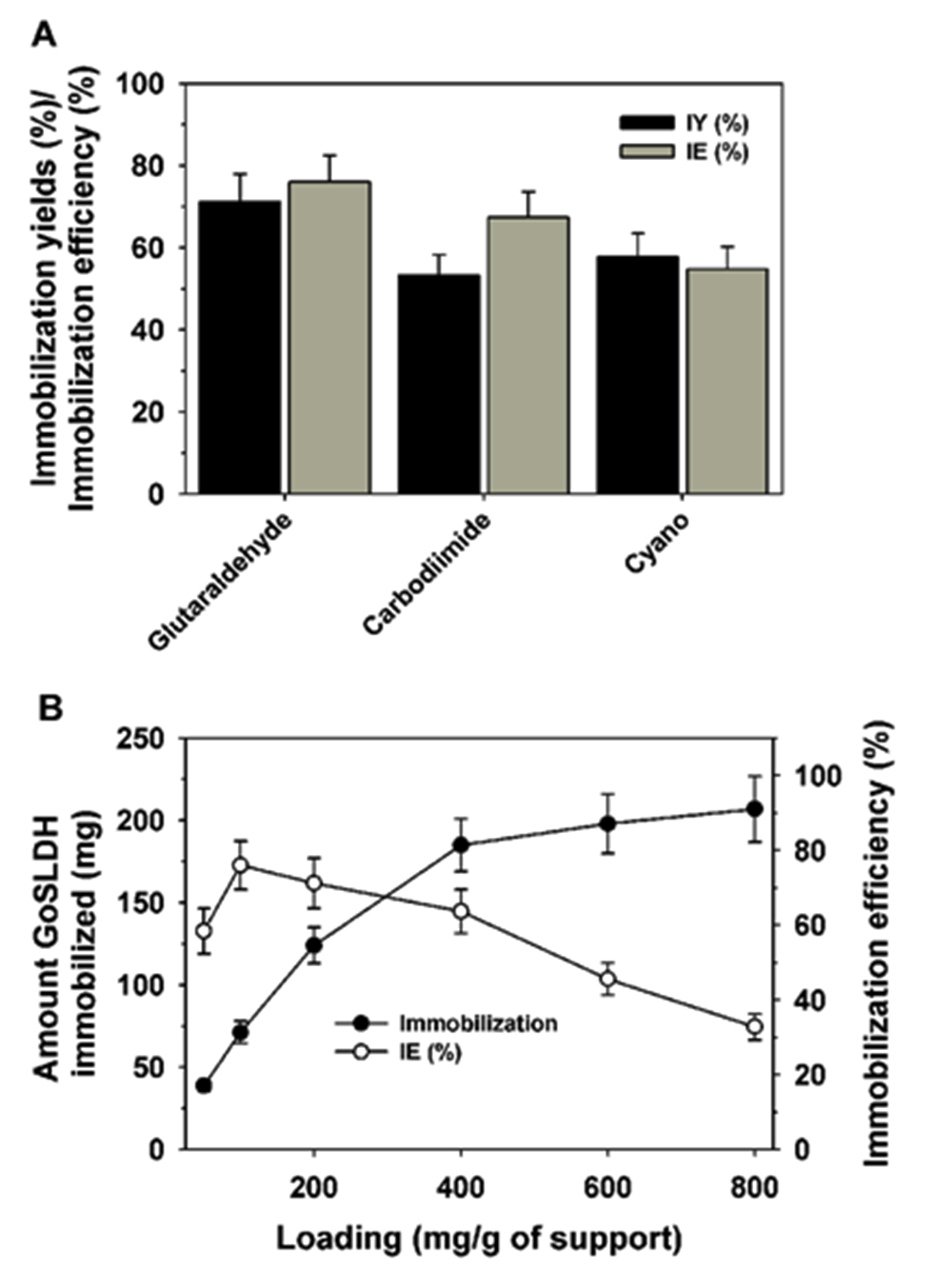
**

**Figure S4.** Immobilization of GoSLDH. Influence of functional groups of SiO_2_ nanoparticles on covalent immobilization (A) and loading on the glutaraldehyde modified SiO_2_ nanoparticles (B).


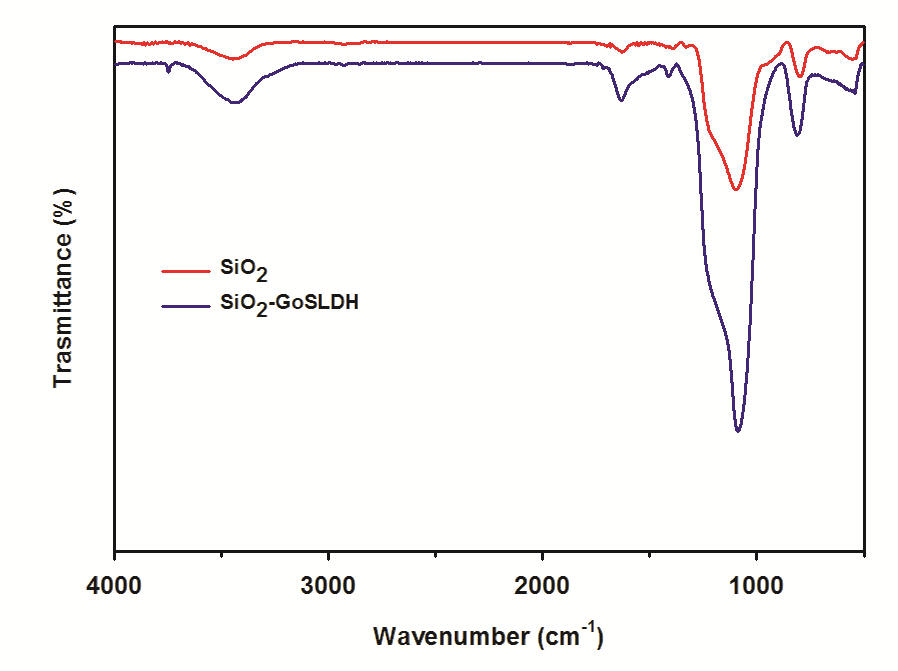


**Figure S5.** FTIR spectra of GoSLDH immobilized on SiO_2_ nano-particles.


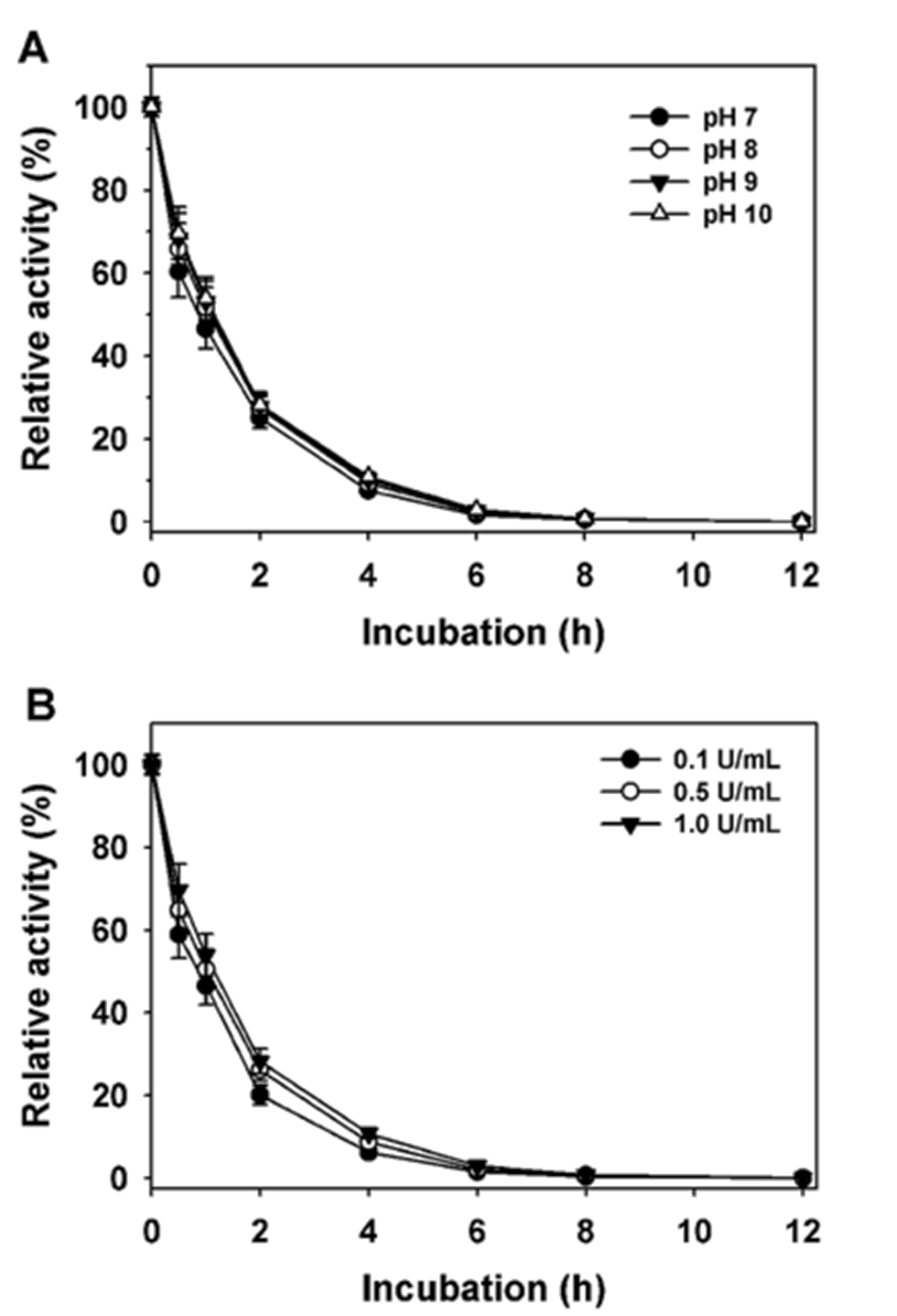


**Figure S6.** Stability of purified GoSLDH at 25 ºC. (A) inactivation courses at different pH values using 1.0 U/mL of enzyme and (B) inactivation courses at pH 10.0 of different enzyme concentrations. The initial activity was considered as 100%.


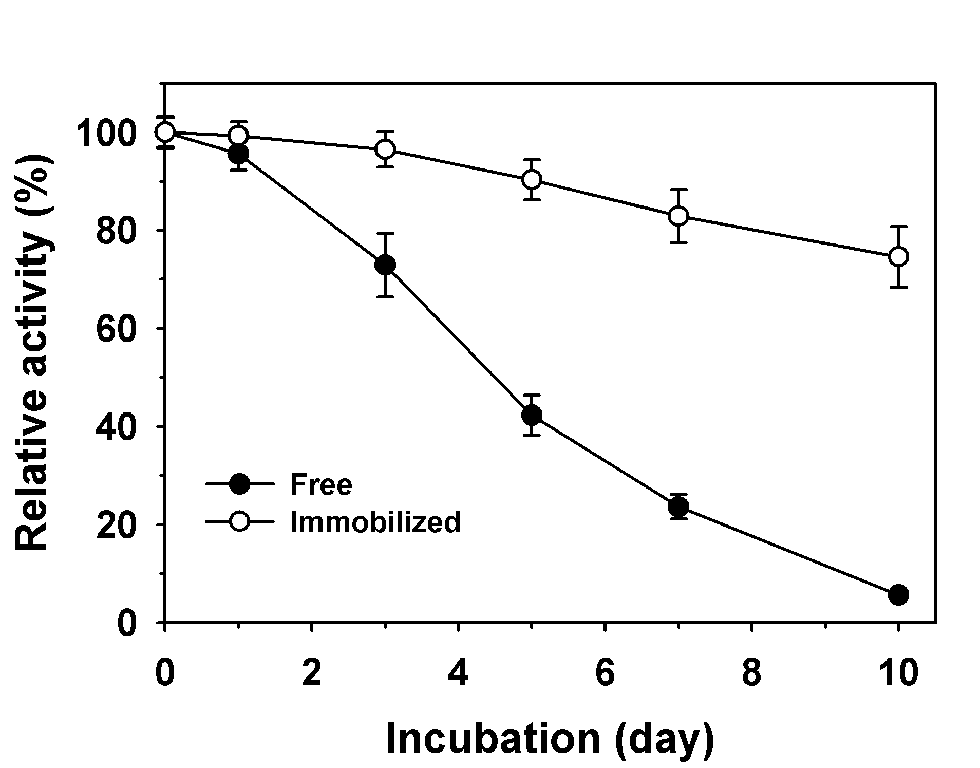


**Figure S7.** Storage stability of GoSLDH at 4 ºC.


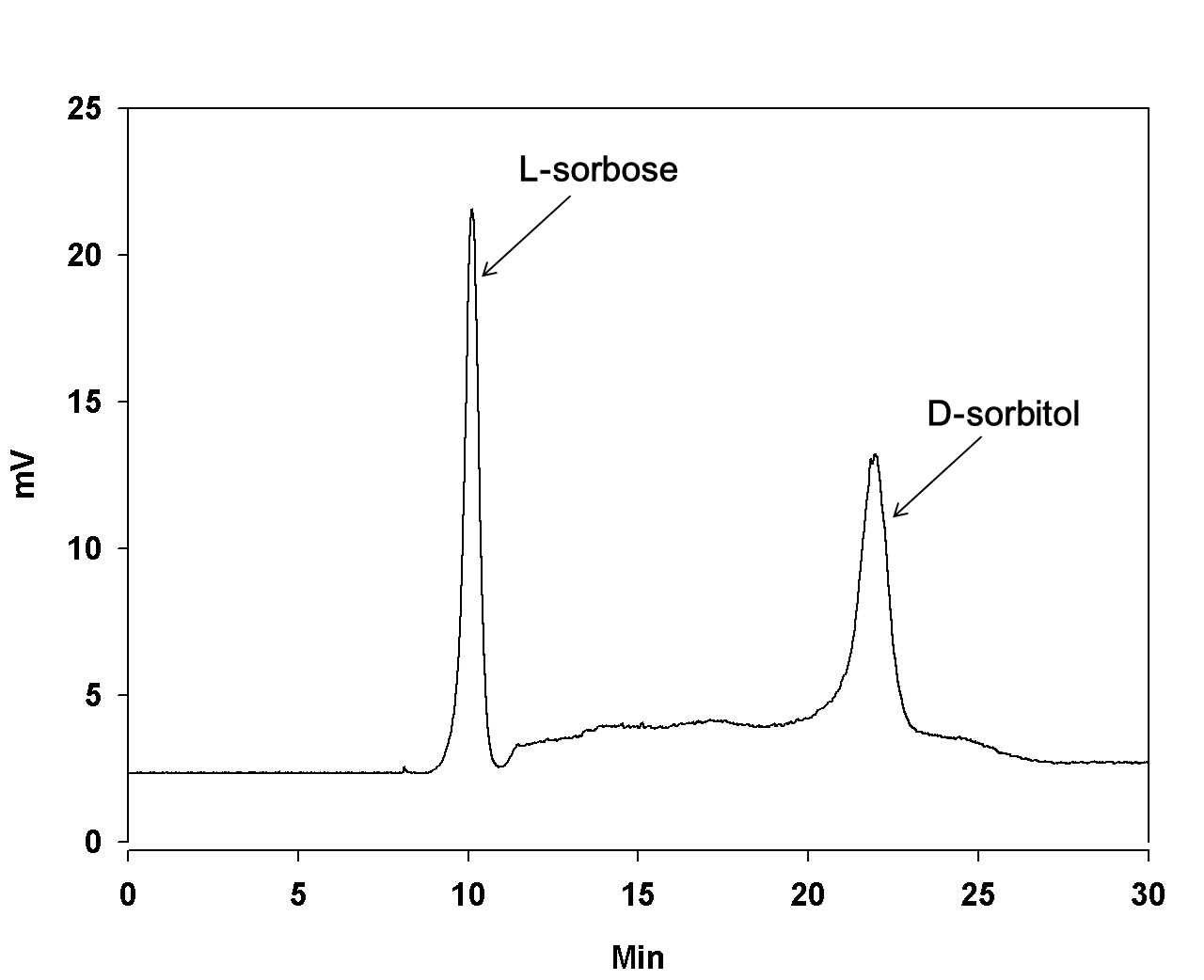


**Figure S8.** HPLC analysis of d-sorbitol oxidation product by GoSLDH.

**Table S1.** Effect of different metal ions on the activity of recombinant GoSLDH.

| Metal ions | Relative activity (%) |
| --- | --- |
| None | 100 ± 4 |
| Mn^2+^ | 103 ± 8 |
| Co^2+^ | 97 ± 4 |
| Mg^2+^ | 91 ± 6 |
| Ca^2+^ | 87 ± 8 |
| K^+^ | 86 ± 4 |
| Ba^2+^ | 80 ± 6 |
| Zn^2+^ | 70 ± 5 |
| Cu^2+^ | 69 ± 6 |

The purified enzyme was assayed in standard conditions with 5 mM metal ions. The activity of GoSLDH measured in the absence of metal ions was considered to be 100%.

**Table S2.** Substrate specificity of purified recombinant GoSLDH.

| Substrate | Specific activity (U/mg protein) |
| --- | --- |
| d-sorbitol | 3570 |
| d-arabitol | 2490 |
| Mannitol | 2130 |
| l-arabinitol | ND |
| Xylitol | ND |
| Ribitol | ND |
| Myo-inositol | ND |
| Glycerol | ND |

Reaction was performed with 200 mM substrates in glycine-NaOH buffer (pH 10) containing NADP^+^ as a coenzyme.

ND: Not detected

**Table S3.** Scoring parameters for docking of d-sorbitol with GoSLDH and PfMDH.

|  | Interactions Overview | H-Bond Acceptor | H- Bond Donor | Distance  (Å) | Docking Score (Kcal/Mol) | Docking energy (Kcal/Mol) | ΔE  (Kcal/Mol) | ΔG_bind_  (Kcal/Mol) |
| --- | --- | --- | --- | --- | --- | --- | --- | --- |
| D-sorbitol with GoSLDH | ASN190(O) O----H (H) | (Li)O–H | (PR)C=O | 1.891 | -8.686 | -56.30 | -28.67 | -28.67 |
|  | ASN190(H) H---O (O) | (PR)N–H | (Li)C–O | 2.357 |  |  |  |  |
|  | ASN190(O) O----H (H) | (Li)O–H | (PR)C=O | 1.693 |  |  |  |  |
|  | LEU191(O) O----H (H) | (Li)O–H | (PR)C=O | 2.133 |  |  |  |  |
|  | LYS195 (H) H----O (O) | (PR)N–H | (Li)C–O | 1.787 |  |  |  |  |
| D-sorbitol with PfMDH | ASN191(O) O----H (H) | (Li)O–H | (PR)C=O | 2.022 | -6.519 | -41.17 | -21.97 | -32.61 |
|  | ASN191 (H)H----O (O) | (PR)N–H | (Li)C–O | 2.421 |  |  |  |  |

**Table S4.** Determination of the denaturation constant (*k*_d_) and half-life (*t*_1/2_) values for the free and immobilized GoSLDH at 25 °C.

| Parameter | GoSLDH | | |
| --- | --- | --- | --- |
|  | Free | Immobilized | Cross-linked |
| *k*_d_ (h^-1^) | 0.591 | 0.100 | 0.044 |
| *r*^2^ | 0.998 | 0.974 | 0.983 |
| *t*_1/2_ (h) | 1.17 | 6.93 | 15.75 |

**Table S5.** Optical rotation analysis of the d-sorbitol oxidation product of GoSLDH by polarimetry.

| [α]^D^ | Specific rotation (^o^) |
| --- | --- |
| d-sorbose | +37.0 to +44.0 |
| l-sorbose | -42.0 to -44.0 |
| d-sorbitol oxidation product of GoSLDH | -43.4 |
